# A common evolutionary origin reveals fundamental principles of protein insertases

**DOI:** 10.1371/journal.pbio.3001558

**Published:** 2022-03-02

**Authors:** F.-Nora Vögtle, Hans-Georg Koch, Chris Meisinger

**Affiliations:** 1 Center for Molecular Biology of Heidelberg University (ZMBH), DKFZ-ZMBH Alliance, Heidelberg, Germany; 2 Network Aging Research, Heidelberg University, Heidelberg, Germany; 3 CIBSS—Centre for Integrative Biological Signalling Studies, University of Freiburg, Freiburg, Germany; 4 Institute of Biochemistry and Molecular Biology, ZBMZ, Faculty of Medicine, University of Freiburg, Freiburg, Germany; 5 BIOSS Centre for Biological Signalling Studies, University of Freiburg, Freiburg, Germany

## Abstract

Membrane proteins require protein machineries to insert their hydrophobic transmembrane domains into the lipid bilayer. This Primer explores the implications of a new PLOS Biology study of protein insertases which reveals that the fundamental mechanism of membrane protein insertion is universally conserved.

## Teaser

Membrane proteins require protein machineries to insert their hydrophobic transmembrane domains (TMDs) into the lipid bilayer. A functional analysis of protein insertases in this issue of *PLOS Biology* reveals that the fundamental mechanism of membrane protein insertion is universally conserved.

The evolvement of complex eukaryotic cells was accompanied by the formation of subcompartments surrounded by lipid bilayers. Confining specific reactions to specialized organelles came at the cost of transporting proteins into or across lipid barriers. Protein insertases are central players in this process and facilitate the correct lipid insertion of client proteins by local distortion and compression of the lipid bilayer. Protein insertases transport unfolded polypeptides and are already present in prokaryotic membranes (YidC and DUF106) [1]. Due to their evolutionary origin, related insertases are present in the inner membrane of mitochondria (Oxa1) and the thylakoid membrane of chloroplasts (Alb3). These so-called Oxa1 family members are closely related and can functionally compensate for each other [2]. Initial bioinformatic analyses revealed remote Oxa1 homologs also in the endoplasmic reticulum (ER), suggesting the existence of an even broader Oxa1 superfamily, consisting of YidC and the 3 ER paralogs TMCO1, EMC3, and GET1 [3–5]. The relationship between these proteins was further supported by structural characterizations of the ER insertases and by comparison with the bacterial YidC [4,6,7]. This led to the hypothesis that the Oxa1 superfamily operates by a common mechanistic principle in all kingdoms of life [4]. The study by Güngör and colleagues in this issue of *PLOS Biology* now validates this hypothesis by demonstrating that the core components of the ER membrane complex (EMC) can functionally replace the mitochondrial Oxa1 insertase [8,9].

**Fig 1:**
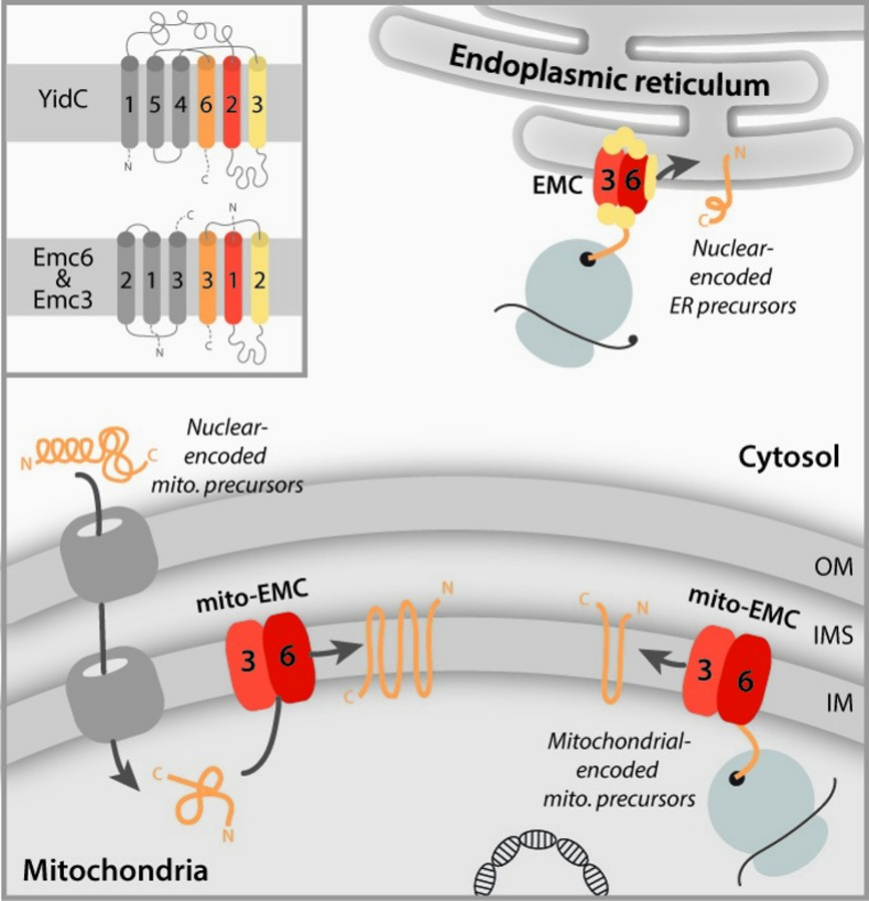
The core members Emc3–Emc6 of the ER insertase EMC can functionally replace the mitochondrial inner membrane insertase Oxa1. Oxa1 mediates insertion of nuclear-encoded precursor proteins that are imported from the cytosol as well as mitochondrial-encoded precursors, which are exported from the matrix side. Both functions were maintained in *oxa1*Δ cells when an Emc3–Emc6 fusion protein was targeted to the inner mitochondrial membrane (mito-EMC). In the inset, the topology of *Escherichia coli* YidC and the EMC subunits Emc6 (left) and Emc3 (right) are shown [4]. Conserved transmembrane helices of the Oxa1 superfamily are highlighted in color. EMC, ER membrane complex; ER, endoplasmic reticulum; IM, inner membrane; IMS, intermembrane space; OM, outer membrane.

The EMC forms an insertase consisting of 8 (yeast) to 9 (mammals) subunits. Cryo-EM structures revealed a striking similarity of the 3 transmembrane domains (TMDs) of Emc3 with other known ER insertases. Furthermore, the 3 TMDs of Emc3 are topologically similar to TMDs 2, 3, and 6 in *Escherichia coli* YidC (TMDs 1, 2, and 5 in YidC of gram-positive bacteria, which consist of only 5 TMDs). Structural and molecular modeling suggests that the rather short TMDs of Emc3 and YidC could execute protein insertion by local thinning of the lipid bilayer [4,10]. This would imply that general destabilization of the membrane is the common mechanism for protein insertion by the Oxa1 superfamily. Emc3 interacts with Emc6, forming the 6 TMD core of the EMC machinery. Güngör and colleagues used a genetically fused Emc6–Emc3 core that resembles the YidC insertase and modified it by addition of an amino-terminal mitochondrial targeting signal and a carboxyl-terminal ribosome-binding site. The construct termed mito-EMC thus combined the TMDs of the EMC core with elements for mitochondrial sublocalization and specific Oxa1 functions (Fig 1). Intriguingly, the expression of mito-EMC partially restored growth of *oxa1Δ* yeast cells even under conditions, when Oxa1-mediated membrane protein insertion is essential.

Oxa1 mediates insertion of mitochondrial- and of nuclear-encoded membrane proteins that use the conservative insertion pathway. In an elegant in organello approach using radiolabeled model substrates and limited proteolysis, the authors verified the correct topological insertion of nuclear-encoded Oxa1 substrates into the inner membrane by mito-EMC. Furthermore, protein insertion by mito-EMC depends like Oxa1-mediated insertion on the negative charge distribution within its substrates, further corroborating a common mechanism for membrane protein insertion by ER and mitochondrial insertases. Membrane integration of mitochondrial-encoded proteins by mito-EMC was assessed via radiolabeling of mitochondrial translation products in combination with carbonate extraction to probe for their membrane integration. Mito-EMC efficiently mediated insertion of the majority of mitochondrial-encoded proteins and restored the endogenous levels of Cox2, an Oxa1-dependent substrate. Intriguingly, a striking difference in synthesis and integration of Atp9 was detected. Immunoprecipitation experiments showed that mito-EMC, in contrast to Oxa1, did not coisolate ATPase subunits. The decameric Atp9 ring was also absent in mito-EMC organelles. Formation of the Atp9 oligomer represents an intermediate in ATPase assembly, and its compromised assembly was also reflected by decreased ATPase activity in mito-EMC–containing mitochondria. In summary, mito-EMC promotes like Oxa1 efficient membrane insertion of nuclear and mitochondrial encoded proteins, but it obviously lacks the assembly function of Oxa1 for the mitochondrial ATPase.

Güngör and colleagues uncovered that the EMC core complex can functionally replace the mitochondrial insertase Oxa1, pointing toward the conservation of a fundamental mechanism despite an evolutionary separation of archaeal and bacterial lineages (which later gave rise to the ER and mitochondria, respectively), which took place about 3 billion years ago. The compensation by mito-EMC is surprising also in regard of the different lipid composition of ER and mitochondria and further supports membrane thinning as mechanism for TMD insertion, which is mainly dependent on lipid chain length rather than overall lipid composition. Intriguingly, mito-EMC failed to rescue the phenotype upon loss of the Oxa1 paralog Cox18. Cox18 cooperates with 2 further mitochondrial proteins, and mito-EMC might not be able to engage in these interactions and therefore cannot compensate for loss of Cox18. Similarly, a missing interaction of mito-EMC with ATPase assembly factors may account for the deficit in Atp9 ring formation and ATPase activity. Mito-EMC consists only of 2 of the 8 EMC subunits present in yeast. It will be interesting to investigate the role of the additional EMC subunits on EMC function and substrate interaction in evolutionary distinct membrane systems.

Taken together, the work by Güngör and colleagues reveals that 2 subunits of the EMC, Emc3 and Emc6, are sufficient to form a minimal insertase that can mediate the insertion of both membrane proteins with simple topologies as well as complex multipass proteins with several TMDs. Furthermore, a particular charge distribution within the translocated substrate protein is required for both EMC- and Oxa1-dependent insertion, further supporting a common and evolutionary conserved insertion mechanism. Together with the recent suggestion that the seemingly unrelated protein transport channels SecY/Sec61 evolved through gene duplication and subsequent fusion from an Oxa1 family member [5], these findings support a common and ancient origin of protein transport systems. Functional investigations like the ones performed by Güngör and colleagues are now allowing the dissection of fundamental principles of membrane protein insertion conserved across all kingdoms of life.

## Abbreviations

EMC: ER membrane complex
ER: endoplasmic reticulum
TMD: transmembrane domain
